# Current Endoscopic Management of Malignant Biliary Stricture

**DOI:** 10.3390/medicina56030114

**Published:** 2020-03-05

**Authors:** Chi-Chih Wang, Tzu-Wei Yang, Wen-Wei Sung, Ming-Chang Tsai

**Affiliations:** 1 Institute of Medicine, Chung Shan Medical University, Taichung 40201, Taiwan; bananaudwang@gmail.com (C.-C.W.); flutewayne@gmail.com (W.-W.S.); 2 School of Medicine, Chung Shan Medical University, Taichung 40201, Taiwan; joviyoung@gmail.com; 3 Division of Gastroenterology and Hepatology, Department of Internal Medicine, Chung Shan Medical University Hospital, Taichung 40201, Taiwan; 4 Institute and Department of Biological Science and Technology, National Chiao Tung University, Hsinchu 30010, Taiwan; 5 Department of Urology, Chung Shan Medical University Hospital, Taichung 40201, Taiwan

**Keywords:** endoscopic management, malignancy, biliary stricture

## Abstract

Biliary and pancreatic cancers occur silently in the initial stage and become unresectable within a short time. When these diseases become symptomatic, biliary obstruction, either with or without infection, occurs frequently due to the anatomy associated with these cancers. The endoscopic management of these patients has changed, both with time and with improvements in medical devices. In this review, we present updated and integrated concepts for the endoscopic management of malignant biliary stricture. Endoscopic biliary drainage had been indicated in malignant biliary obstruction, but the concept of endoscopic management has changed with time. Although routine endoscopic stenting should not be performed in resectable malignant distal biliary obstruction (MDBO) patients, endoscopic biliary drainage is the treatment of choice for palliation in unresectable MDBO patients. Self-expanding metal stents (SEMS) have better stent patency and lower costs compared with plastic stents (PS). For malignant hilum obstruction, PS and uncovered SEMS yield similar short-term outcomes, while a covered stent is not usually used due to a potential unintentional obstruction of contralateral ducts.

## 1. Introduction

Percutaneous transhepatic biliary drainage was introduced ~40 years ago [1] for the treatment of obstructive jaundice; however, the more convenient endoscopic trans-papillary drainage, which was introduced in 1981 [2,3], is more commonly used in clinical practice today. The most common malignancy diseases that can cause obstructive jaundice are cholangiocarcinoma, pancreatic cancer [4], and ampullary neoplasms. Cholangiocarcinoma, which arises from the epithelial cells of the intrahepatic or extrahepatic bile ducts, can be divided into intrahepatic cholangiocarcinoma (ICC) and extrahepatic cholangiocarcinoma (ECC). The incidence of ECC has increased in the USA [5], whereas ICC incidence has increased in both sexes in Europe [6]. At the same time, the pancreatic cancer burden has also increased in recent years [7], making endoscopic management of malignant obstructive jaundice an important issue. Due to the lack of effective treatment choice, the need for endoscopic/radiologic approaches has increased and the clinical condition may be changed due to emerging evidence in targeted therapies of cholangiocarcinoma in the following years [8].

Differences in the level of obstruction of the biliary system allow for a further division of these kinds of problems into resectable or unresectable hilum or distal biliary obstructions. Computed tomography (CT), magnetic resonance imaging (MRI), magnetic resonance cholangiopancreatography (MRCP), and even endoscopic ultrasound sonography (EUS) should be used to evaluate the stage and primary source of a malignancy [9,10].

## 2. Resectable Malignancy

Treatment plans for malignancies with different primary origins differ from one another when resectable biliary obstructive malignancy diseases are discussed. In general, routine endoscopic stenting has no obvious clinical benefits for patients with malignant distal biliary obstruction (MDBO) [11,12,13,14]. Nevertheless, most endoscopists worldwide still perform routine endoscopic retrograde cholangiopancreatography (ERCP) and even biliary stenting prior to surgical approaches.

**Figure 1:**
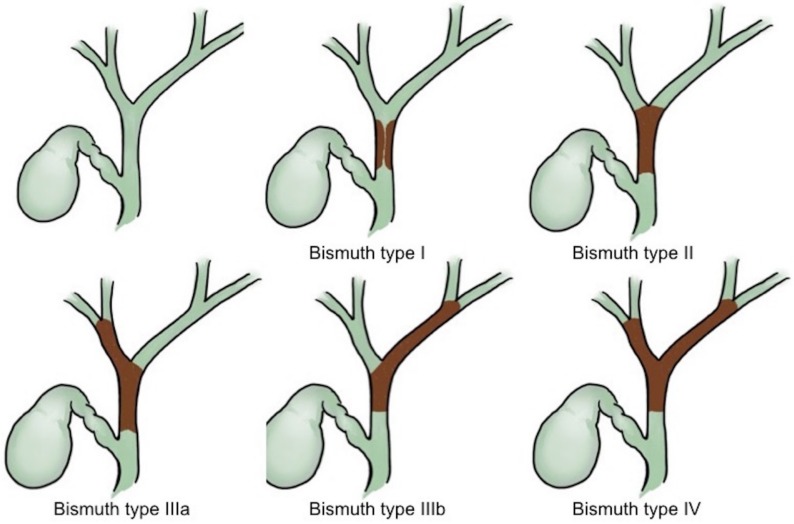
Bismuth classifications of cholangiocarcinoma.

For cholangiocarcinoma, the Bismuth classifications (Figure 1) have been used for decades as a treatment guide [15]. ERCP is useful for confirmation of the obstruction level and for histological proof via either brushing cytology or biopsy, although these have limited sensitivity [16,17]. Mother–baby cholangioscopy [18,19,20] and single-operator SpyGlass cholangioscopy [21] plus targeted biopsy can improve the sensitivity for detecting a biliary malignancy to 89–100% and 66.2% and the specificity to 87–96% and 97.0%, respectively. For Bismuth type I lesions without lymph node and distant metastasis, surgical resection should be considered a first approach.

For hilum cholangiocarcinoma without distant metastasis, resectable tumors present the possibility of resection of the involved intra- and extrahepatic bile ducts as well as the associated hepatic lobes and caudate lobe. Some recent reports have shown excellent results after surgical resection in patients with Bismuth III and IV disease [22,23], but the surgical management of these patients is still under debate. 

Endoscopic stenting is the mainstream endoscopic management approach for malignant distal biliary obstruction of cholangiocarcinoma or pancreatic cancers, but most clinical studies [11,24] and meta-analyses [12,13,14] have failed to show any benefits of routine pre-operative endoscopic stenting in MDBO patients. However, although this stenting should be avoided in resectable MDBO, an interesting study showed that more than 80% of doctors in academic centers in the USA still perform this drainage prior to surgery [25]. In pancreatic cancer patients with asymptomatic obstructive jaundice, the recommendation of the Standards of Practice Committee of the American Society for Gastrointestinal Endoscopy (ASGE) is against routine preoperative ERCP [9].

In ampullary neoplasms, EUS and intraductal ultrasound sonography (IDUS) can assess the depth of invasion as well as the intraductal extension. Importantly, surgical ampullectomy can reduce medical expenses yet have similar clinical outcomes for those that are achieved via pancreaticoduodenectomy [26] for ampullary neoplasms that are limited to the ampulla of Vater. Endoscopic ampullectomy [27] can be performed for adenoma and early ampullary adenocarcinoma by experienced endoscopists in well-equipped endoscopy centers.

## 3. Unresectable Malignancy

### 3.1. Malignant Distal Biliary Obstruction

Endoscopic biliary drainage with biliary stent placement is the treatment of choice for palliation in patients with distal malignant biliary obstruction that is caused by unresectable neoplasms, with a success rate of up to 95% [28,29,30] and with a lower morbidity compared to surgery [31]. Endoscopic drainage has a low risk of complications, but it is associated with a high probability of recurrent biliary obstruction [32] when compared with surgical bypass. After an endobiliary radiofrequency ablation (RFA) system was applied to biliary malignant obstructions, series intra-luminal RFA and self-expanding metal stents (SEMS) became a safe treatment choice with better intervention-free survival compared to SEMS alone [33,34,35]. Percutaneous trans-hepatic cholangiography and drainage (PTCD) should be reserved for unsuccessful endoscopic procedures in patients with MDBO. 

In some situations, PTCD has been used as part of a rendezvous endoscopic approach. A comparison in 2016 between EUS-guided and PTCD rendezvous drainage after the failure of primary ERCP in MDBO showed that EUS rendezvous had a significantly lower success rate than the PTCD rendezvous [36]. However, successful EUS rendezvous offered a significantly shorter post-procedure hospital stay and fewer follow-up biliary interventions. As technology improves and skills in EUS-guided biliary drainage gradually mature, EUS-guided biliary drainage will produce better outcomes than that of conventional ERCP biliary drainage in high-tech endoscopy centers [37,38,39].

### 3.2. Stent Selection

**Figure 2:**
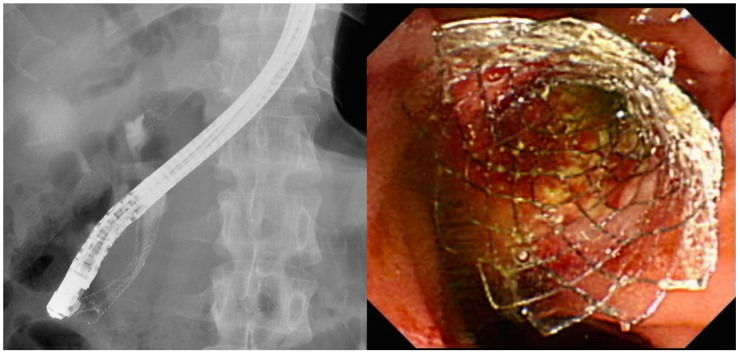
Self-expanding metal stents for use in pancreatic cancers with malignant distal biliary obstruction.

**Figure 3:**
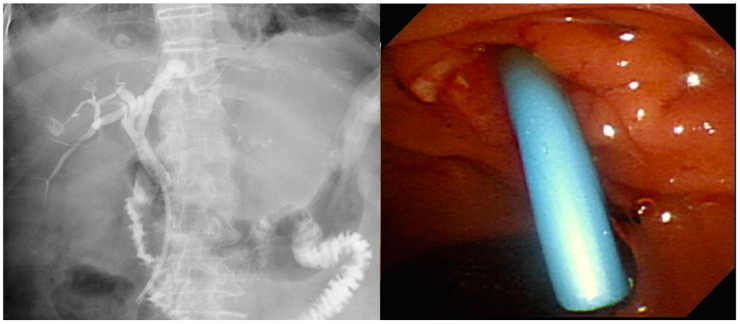
Plastic stent for use in pancreatic cancers with malignant distal biliary obstruction.

Different stent types are available for the treatment of MDBO. SEMS (Figure 2) have a larger luminal diameter than plastic stents (Figure 3), and they were designed to overcome the limitations of occlusion and stent patency that are associated with plastic stents [40]. SEMS offer a better stent patency of 3.6–9.1 months compared to 1.8–5.5 months for plastic stents [41,42,43,44] in MDBO, but the median patient survival times are similar with either stent type [45]. SEMS are more expensive than plastic stents, but SEMS lower the total medical expenses [41,46] due to the reduced frequency of re-interventions. Different types of metal stents are now available, and covered stents show superior stent patency over uncovered metal stents in the treatment of MDBO [47]. Some previous case studies showed no significant differences in stent patency between covered and uncovered SEMS because covered stents had more drawbacks due either to stent migration or the occurrence of acute cholecystitis [48,49,50]. Recent studies have revealed that fully covered SEMS (FCSEMS) that are used for MDBO treatment have low stent migration rates and trigger few cholecystitis events [51]. A prospective study of unresectable pancreatic cancer with obstructive jaundice demonstrated a significantly longer survival time and no stent dysfunction for covered SEMS with an anti-migration system than it did without an anti-migration system [52,53]. These studies suggest that covered SEMS are a valuable and cost-effective option for MDBO treatment because of their increased patency, less tumor in-growth, and easy removal [53,54]. However, several recent large studies [55,56,57] and a recent meta-analysis from Canada [58] have revealed no clear benefit of FCSEMS over uncovered SEMS (UCSEMS) in terms of stent patency and complications. Conversely, one multi-center study from Italy showed increased stent migration and even earlier stent occlusion with FCSEMS [59], while two other studies revealed no stent patency benefit between partially covered SEMS (PCSEMS) and UCSEMS [60,61]. Most studies to date on MDBO have shown no significant differences in stent patency between FCSEMS, PCSMES, or UCSEMS. Notably, while FCSEMS is used by many well-recognized endoscopists, its superiority is still under debate.

## 4. Malignant Hilar Biliary Obstruction

EUS is a valuable diagnostic modality for the staging of hilum cholangiocarcinomas, particularly for the evaluation of unresectable perihilar cholangiocarcinoma for liver transplantation [62]. Percutaneous laparoscopic biopsy and even EUS-fine-needle aspiration (EUS-FNA) of the primary tumor are not recommended in patients who are candidates for liver transplantation because of the high risk of peritoneal dissemination following these procedures [63]. Endoscopic biliary stenting for malignant obstructive jaundice offers symptomatic improvements [64,65] and has been widely accepted as an effective palliation treatment [31,66], but no widespread consensus has yet been reached regarding the optimal stent types and placement procedures for stent insertion [67]. Liver volume drainage in excess of 50% should be achieved using multiple stenting to improve survival [68]. Selection of the drainage method and stent requires the use of CT, MRI, or MRCP. 

### 4.1. Bilateral or Unilateral Drainage

Some evidence indicates that in cases of bilateral biliary opacification, bilateral drainage offers a better survival benefit when compared to unilateral drainage [69,70]. After adjustment for the Bismuth stage, two parallel SEMS should be deployed for advanced cholangiocarcinomas such as Bismuth types III and IV obstructions [71]. PTCD is considered an alternative choice if primary endoscopic drainage fails, and some evidence has shown that PTCD is an even better choice for advanced hilum obstruction, which occurs in Bismuth types III and IV patients with unresectable malignant obstructions [71,72,73,74].

**Figure 4:**
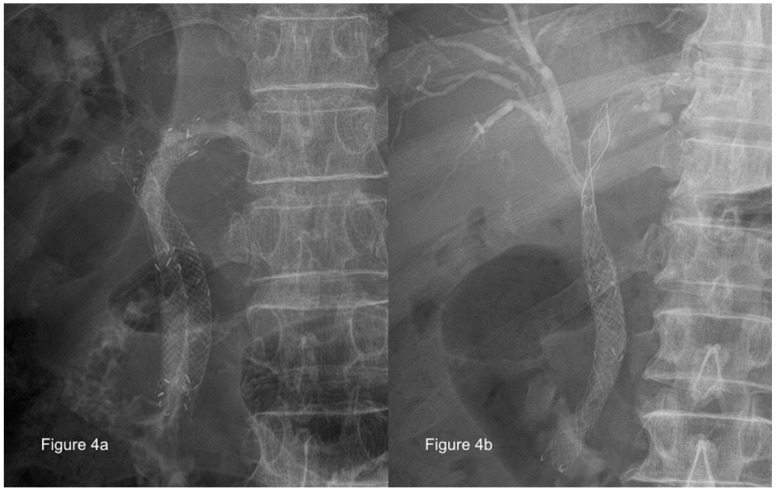
Parallel or stent-in-stent deployment of self-expanding metal stents for use in treatment of malignant hilar biliary obstruction (Courtesy of Dr. Nai-Jen Liu). (**a**) Parallel deployment; (**b**) stent-in-stent deployment

The evidence is still insufficient and no clear consensus has been reached regarding the benefits of unilateral versus bilateral drainage for hilar malignant obstruction, although the bilateral approach can be used by most experts using the SEMS stent-within-stent placement [75,76] with the newly designed Y-shaped devices, and this shows promising results [77,78]. These procedures can be performed using parallel [79] (Figure 4a) or stent-in-stent [80] (Figure 4b) deployment of SEMS, but not enough evidence is available to identify which method is preferable. Previous data have shown that draining 25% of the liver volume was sufficient to relieve jaundice [81]; however, a recent study indicated that patients who had liver volume drainage of >50% experienced greater jaundice relief than those who had a lower volume drained [68]. However, because the right lobe of the liver covers 55–60% of the liver volume, while the left and caudate lobes cover 30–35% and 10% of the liver volume, respectively [82], draining >50% of the liver volume usually requires the use of bilateral stenting or multi-segmental stenting, depending on the individual patient’s anatomy. 

### 4.2. Stent Selection

Plastic stents and uncovered SEMS yield similar short-term outcomes in patients with malignant hilar strictures due to stent migration in SEMS [83]. SEMS provide a longer biliary patency when compared with plastic stents; thus, they are the choice of most endoscopists [64,69,74,84]. Covered stents are not usually used in patients with malignant hilar biliary obstruction due to the possibility of unintentional obstruction of the contralateral and/or side branch ducts. 

### 4.3. Rescue Management

EUS-guided biliary drainage via EUS-guided hepaticogastrostomy (EUS-HGS) is a good alternative for draining malignant hilar biliary obstruction following the failure of an initial ERCP. An EUS-guided rendezvous procedure with the conventional ERCP access [85,86] is typically used in most situations, except for concurrent duodenal obstruction. EUS-HGS [87,88] is the preferred treatment option if either a guidewire cannot pass through the duodenum or the presence of a concurrent duodenal obstruction is found. Another alternative approach to EUS-HGS is EUS-guided choledochoduodenostomy (EUS-CDS) [89], which offers management methods other than PTCD. Due to the high rate of complications, EUS-guided biliary drainage has served as an alternative approach over the last two years; however, some encouraging results have shown a similar safety profile and procedure success rate and even better stent patency during that same timeframe [36,37,90].

## 5. Summary

In cases of malignant biliary obstruction, CT, MRI, MRCP, and EUS should be used for tumor staging and further drainage planning. For resectable malignant biliary obstruction lesions, routine endoscopic drainage or ERCP are not advised, except in cases of concurrent infection. Endoscopic drainage is the treatment of choice for unresectable biliary obstructions. PTCD and EUS-guided biliary drainage can serve as alternative approach methods. EUS-guided biliary drainage can provide equal clinical benefits without increasing complications in a high-tech endoscopy center. In addition, PTCD plays an important role in community hospitals, where the ERCP and/or EUS approaches are not available.

**Figure 5:**
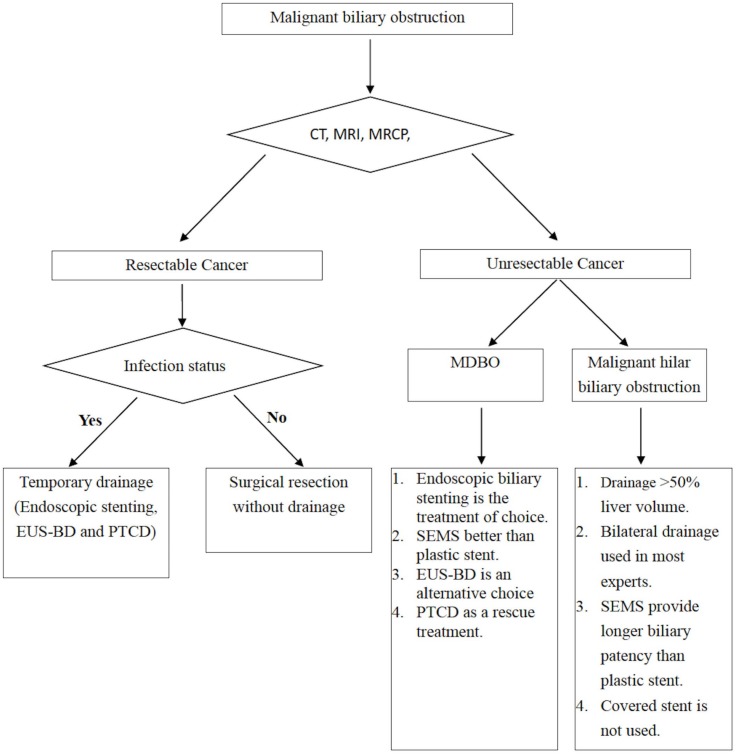
Flow chart for the management of malignant biliary obstruction.

For MDBO, single-stent insertion is adequate, and SEMS have better patency than plastic stents; however, the superiority of FCSEMS, PCSEMS, and UCSEMS remains under debate. For malignant hilar biliary obstructions, drainage of >50% of the liver volume should be achieved by either bilateral stenting or multi-segmental stenting, and SEMS’ longer biliary patency when compared with plastic stents should be considered. The general approaches to malignant biliary obstruction are summarized in the flow chart presented in Figure 5.

## Abbreviations

ASGE: American Society for Gastrointestinal Endoscopy; CT: computed tomography; ECC: extrahepatic cholangiocarcinoma; ERCP: endoscopic retrograde cholangiopancreatography; EUS: endoscopic ultrasound sonography; EUS-FNA: endoscopic ultrasound sonography–fine-needle aspiration; EUS-BD: endoscopic ultrasound-guided biliary drainage; FCSEMS: fully covered self-expanding metal stent; ICC: intrahepatic cholangiocarcinoma; IDUS: intraductal ultrasound sonography; MDBO: malignant distal biliary obstruction; MRCP: magnetic resonance cholangiopancreatography; MRI: magnetic resonance imaging; PCSEMS: partially covered self-expanding metal stent; PS: plastic stent; PTCD: percutaneous trans-hepatic cholangiography and drainage; SEMS: self-expanding metal stent; UCSEMS: uncovered self-expanding metal stent.
